# The mutational signatures of cancer: can passengers set a direction for prognosis?

**DOI:** 10.18632/oncoscience.588

**Published:** 2023-10-02

**Authors:** Peeter Karihtala

**Keywords:** cancer, COSMIC, mortality, mutational signatures, survival

In the early days of cancer genomics, the field concentrated mostly to find actively cancer-progressing driver mutations. After the rapid development of modern sequencing technologies in the early 2010s, it was noted that passenger mutations could not be just random, irrelevant debris, but rather scars that have occurred during underlying biological processes of the tumor development and could therefore represent a historical record of carcinogenesis [1, 2]. This paved the way for the mutational signatures, the concept which is nowadays defined as characteristic patterns of somatic mutations that occur in cancer genomes [3].

Despite multiple other ways to characterize mutational signatures, the COSMIC (Catalog of Somatic Mutations in Cancer) signatures are extensively studied and recognized as one of the most comprehensive and clinically relevant collections of mutational signatures to date [3]. For example, specific COSMIC single-base substitution (SBS) mutational signatures may tell defects in specific DNA proofreading mechanisms (e.g., SBS10), exposure to specific chemotherapies (e.g., SBS17), or they may be secondary to smoking (e.g., SBS4) [3, 4]. Still, the association of the mutational signatures with prognosis have not been elucidated until recently.

In our two recent papers [5, 6], we used publicly available data from the Cancer Genome Atlas (TCGA) and Pan-Cancer Analysis of Whole Genomes (PCAWG) databases to evaluate if the presence of some COSMIC mutational signatures would be able to improve the prognostic value over the traditional prognostic factors in gastrointestinal or urological cancers, altogether in 13 histological types of cancers. As a common factor in both gastrointestinal and urological cancers, their prognosis varies a lot between and within the tumor types and there is also a lack of established prognostic factors in addition to the TNM stage and histological subtype. While some cancer types and most signatures did not have any association with four different survival endpoints we used, there was e.g., tremendously improved overall and cancer-specific survival in the rectal adenocarcinoma patients with high age-related SBS5 and SBS40 activity. In multivariate analyses, the hazard ratio estimates (for SBS5 0.13; 95% confidence interval 0.03–0.56 and for SBS40 0.072; 95% confidence interval 0.012–0.44) from these analyses exceeded by far the traditional colorectal cancer prognostic factors, including stage. As another example, in the patients with left-sided (but not right-sided) colon adenocarcinoma, the high activity of SBS2 signatures, formed due to APOBEC activity, predicted shortened overall survival.

Certain COSMIC signatures provided relevant prognostic information also in urological cancers, with a significant proportion of the prognostic signatures relating to the activation of APOBEC enzymes (neoplasia). This was observed especially in papillary renal cell carcinomas (pRCC), in tumor type, where other reliable prognostic factors than the stage have been virtually absent. In the time-dependent integrated area under the curve model, a high number of SBS45 signatures yielded values up to 0.93 for predicting improved pRCC-specific survival. Furthermore, APOBEC-related signature SBS2 was associated with improved overall survival and disease-specific survival in bladder carcinomas in the multivariate analysis, while the clock-like signature SBS1 predicted shortened disease-specific survival and progression-free interval in clear cell renal cell carcinomas.

We also compared the expression of individual genes between the low-signature and high-signature groups to gain insight into the potential molecular mechanisms connected to mutational signatures and survival. As an example of the novel findings, the prominent upregulation of the genes belonging to the melanoma antigen (MAGE) family were highly upregulated in the signatures, which predicted poor survival. Again these genes were downregulated in signatures associating with improved outcomes (e.g., SBS45 in pRCC). The MAGE genes are highly conserved in all eukaryotes, and they play a crucial role in adaptation against environmental stress [7] and based on our results, they may be the key drivers of the prognostic role of certain mutational signatures. With using only selected MAGE genes we were also able to provide prognostic information near to that of mutational signatures and clinical variables combined for pRCC patients.

There are currently several obstacles that hinder mutational signatures to be used in clinical practice. Firstly, the available results are still from retrospective databases, although TCGA is one of the largest, most validated and most comprehensive publicly available cancer genomics datasets. Secondly, whole-exome (or whole-genome) sequencing is presently performed only to a minority of the cancer patients and to convert this data to COSMIC mutational signatures requires some bioinformatic skills. However, the use of WES is likely to increase in clinical practice in the near future as the novel personalized treatments emerge and more accurate and cheaper sequencing methods become more widely used.

Based on the accumulating evidence, mutational signatures are not only genomic noise of passenger mutations, but they provide etiological and biological information on carcinogenesis. Clinically more importantly, mutational signatures have also potential to elaborate our understanding on cancer prognosis in specific solid cancers in addition to the traditional prognostic factors. Optimally this could lead to the tailoring of adjuvant treatments and surveillance in the future.
